# Utility of Sepsis Biomarkers and the Infection Probability Score to Discriminate Sepsis and Systemic Inflammatory Response Syndrome in Standard Care Patients

**DOI:** 10.1371/journal.pone.0082946

**Published:** 2013-12-11

**Authors:** Franz Ratzinger, Michael Schuardt, Katherina Eichbichler, Irene Tsirkinidou, Marlene Bauer, Helmuth Haslacher, Dieter Mitteregger, Michael Binder, Heinz Burgmann

**Affiliations:** 1 Department of Laboratory Medicine, Division of Medical and Chemical Laboratory Diagnostics, Medical University of Vienna, Vienna, Austria; 2 Department of Medicine I, Division of Infectious Diseases and Tropical Medicine, Medical University of Vienna, Vienna, Austria; 3 Department of Laboratory Medicine, Division of Clinical Microbiology, Medical University of Vienna, Vienna, Austria; 4 Department of Dermatology, Division of General Dermatology, Medical University of Vienna, Vienna, Austria; Kliniken der Stadt Köln gGmbH, Germany

## Abstract

Physicians are regularly faced with severely ill patients at risk of developing infections. In literature, standard care wards are often neglected, although their patients frequently suffer from a systemic inflammatory response syndrome (SIRS) of unknown origin. Fast identification of patients with infections is vital, as they immediately require appropriate therapy. Further, tools with a high negative predictive value (NPV) to exclude infection or bacteremia are important to increase the cost effectiveness of microbiological examinations and to avoid inappropriate antibiotic treatment. In this prospective cohort study, 2,384 patients with suspected infections were screened for suffering from two or more SIRS criteria on standard care wards. The infection probability score (IPS) and sepsis biomarkers with discriminatory power were assessed regarding their capacity to identify infection or bacteremia. In this cohort finally consisting of 298 SIRS-patients, the infection prevalence was 72%. Bacteremia was found in 25% of cases. For the prediction of infection, the IPS yielded 0.51 ROC-AUC (30.1% sensitivity, 64.6% specificity). Among sepsis biomarkers, lipopolysaccharide binding protein (LBP) was the best parameter with 0.63 ROC-AUC (57.5% sensitivity, 67.1% specificity). For the prediction of bacteremia, the IPS performed slightly better with a ROC-AUC of 0.58 (21.3% sensitivity, 65% specificity). Procalcitonin was the best discriminator with 0.78 ROC-AUC, 86.3% sensitivity, 59.6% specificity and 92.9% NPV. Furthermore, bilirubin and LBP (ROC-AUC: 0.65, 0.62) might also be considered as useful parameters. In summary, the IPS and widely used infection parameters, including CRP or WBC, yielded a poor diagnostic performance for the detection of infection or bacteremia. Additional sepsis biomarkers do not aid in discriminating inflammation from infection. For the prediction of bacteremia procalcitonin, and bilirubin were the most promising parameters, which might be used as a rule for when to take blood cultures or using nucleic acid amplification tests for microbiological diagnostics.

## Introduction

Systemic inflammatory response syndrome (SIRS) is defined as an acute host reaction to various different stimuli, including both infectious and non-infectious causes. The definition of SIRS is based on physiological parameters including body temperature, heart beat rate, respiration rate (or oxygen saturation), as well as abnormalities in leukocyte counts (leukocytosis, an elevation of immature neutrophils or leukopenia) [1]. These criteria are easily applicable but also imply patients without major inflammatory disorders and are therefore not specific. In clinical routine it is of crucial importance to rapidly identify patients with SIRS due to infection (sepsis), as these patients require prompt appropriate management, as well as immediate antimicrobial therapy [2]. On the other hand, improper use of antibiotics in the hospital setting may favor the emergence of multi-resistant bacteria and may be associated with adverse drug reactions resulting in prolonged hospitalization and decreased cost efficiency [3,4,5].

On the basis of clinical criteria alone it is impossible to discriminate between septic patients and patients with SIRS due to other causes. Today, physicians often rely on classical microbiological methods, e.g. blood cultures, to identify possible infection sources. These methods, however, may need several days before results are gained. In contrast, molecular microbiological methods may provide results within hours, but require high amounts of financial as well as laboratory resources. Further, only a limited spectrum of pathogens can be detected by some of these methods. Regardless of the method used, even negative results do not exclude severe infection. In the literature, the true positive rate of blood cultures is ranked between 5–10% and a further five percent are false positives due to contamination [6,7,8]. The costs of unnecessary blood culture requests, especially when false positive are included, are substantial [9,10].

To identify infection in patients with SIRS, various studies have been performed evaluating different assessment scores or laboratory parameters. Among assessment scores, the infection probability score (IPS, range: 0–26 points) represents a prospectively evaluated score with a high negative predictive value (NPV) with which to exclude infection in severely ill patients [11]. This score is calculated using six parameters, namely heart beat rate, respiration rate, body temperature, white blood cell count (WBC), C-reactive protein (CRP), and the sequential organ failure assessment (SOFA) score [12]. Laboratory parameters in use for the rapid identification of infection include procalcitonin (PCT), interleukin 6 (IL-6), lipopolysaccharide binding protein (LBP), and CRP [13,14,15,16]. However, the clinical use of these parameters might be limited, since in literature reports on the diagnostic value of the discrimination of sepsis and SIRS vary. Additionally, assessment scores as well as sepsis parameters have been mainly evaluated in patients requiring intensive care or at emergency departments [15,16,17,18]. Data on the utility of such scores or sepsis parameters in standard care patients presenting with SIRS are rare or not available.

Thus, the present study was set out to assess the utility of the IPS and several sepsis parameters for identifying infections in standard care patients with SIRS.

## Materials and Methods

### Study design and endpoints

Between July 2011 and March 2012, a prospective single-center cohort study was performed at the Vienna General Hospital, Austria, a 2116-bed university hospital. Patients from 27 different standard care wards (14 medical and 13 surgical wards) with clinical suspicion of bacterial infection and for whom blood culture was requested were screened for the following inclusion criteria: two or more SIRS criteria (according to the criteria of the ACCP/SCCM consensus conference [1]), age greater than or equal to 18 years, and the ability to give consent. Iatrogenic neutropenia in patients with malignancies was not considered as a valid SIRS criterion. Exclusion criteria for participation in the study were as follows: surgery within 72 hours prior to the blood culture request (postoperative fever), infection with HIV, fungi or parasites, or inability to assign the patient into an outcome group.

Bacteremia was defined as a positive blood culture result or the detection of bacterial DNA in EDTA plasma for a recognized pathogen. Likewise, to reduce the number of false positive results, coagulase-negative staphylococci (CNS) were regarded as blood stream pathogens only when detected in blood samples drawn on separate occasions [19,20]. After hospital discharge, infection was assessed and classified by the application of the definition criteria of the European Centre of Disease Control (ECDC), which was established for point prevalence studies on hospital-acquired infections [21]. These criteria contain clinical information and microbiological results, as well as laboratory and radiological data. Criteria for the classification of patients with SIRS, due to non-infectious causes, were not found in literature.

### Data collection

Clinical data was collected at the time of study enrollment and after hospital discharge from the individual medical chart. Blood was cultured in a set of blood culture bottles, FA Plus (aerobic) and FN Plus (anaerobic), in the BacT/ALERT 3D automated blood culture system (bioMérieux, Marcy l'Etoile, France). Detection of microbial DNA in blood samples was performed with the LifeCycler® SeptiFast test MGRADE (Hoffmann-La Roche Ltd, Basel Switzerland). The IPS and biomarkers for sepsis were gathered within 18 hours after the initial blood culture request. Patients with an IPS of more than 14 points were considered positive for infection. The following laboratory parameters were used: CRP (Latex test, Beckman Coulter, Brea, USA; lower limit of quantification (LLOQ): 0.04 mg/dl), PCT and IL-6 (both, Hoffmann-La Roche Ltd; LLOQ: 0.03 ng/ml and 1.6 pg/ml, respectively), LBP (IMMULITE 2000 Immunoassay System, Siemens Healthcare, Erlangen Germany; LLOQ: 0.8 µg/ml), bilirubin (Hoffmann-La Roche Ltd; LLOQ: 0.11 mg/dl), and WBC (Stromatolyser-4DS, Sysmex, Norderstedt, Germany; LLOQ: not provided). All laboratory work was performed at an ISO 9001:2008 certified and EN ISO 15189:2008 accredited medical laboratory.

### Ethical issues, anonymization, and data security

The study was approved by the ethics committee of the Medical University of Vienna (EC-No. 518/2011) and was carried out in accordance with the guidelines of the Declaration of Helsinki (1964), including current revisions, and the rules of Good Clinical Practice of the European Commission. Prior to enrollment, patients were informed in detail about the trial and signed a consent form to confirm their participation. To ensure anonymity, every participant was consecutively assigned an identification number, which was used for further analysis. Additional anonymous clinical information and raw data can be requested from the corresponding author.

### Statistical analysis

Continuous data is presented as median and quartiles (Q_1_, Q_3_), categorical data as counts and percentages. Data was statistically analyzed using non-parametric tests, including the Pearson´s χ^2^-test and the Mann-Whitney U test. Furthermore, receiver operating characteristic (ROC) curves of the parameters investigated were drawn to compute the area under the curve (AUC). The DeLong test was used to compare ROC-AUCs of different parameters. To set an optimal cut-off value for optimal differentiation, the Youden-index method was applied. For dichotomized parameters, sensitivity, specificity, negative predictive value (NPV), and positive predictive value (PPV) were calculated and given with 95% confidence intervals. Statistical significance was defined at a p-value less than 0.05 (two-tailed). When appropriate, accumulation of the α-error probability related to multiple testing was corrected using the Bonferroni-Holm method. All calculations were done using SPSS 21.0 (IBM, Hercules, USA) and MedCalc 12.7.0 (MedCalc Software bvba, Ostend, Belgium).

## Results

**Figure 1:**
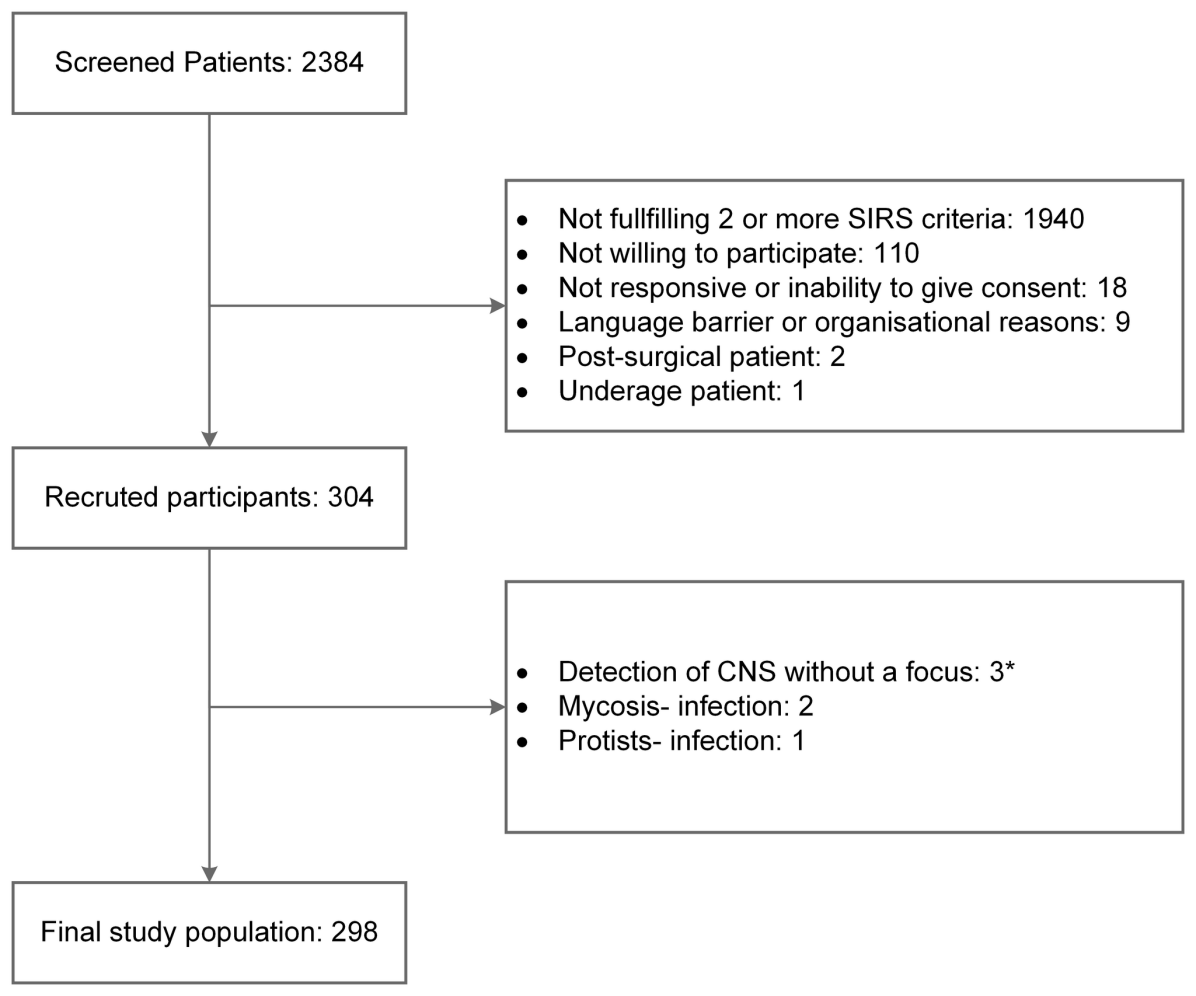
Recruiting of the study population. CNS = coagulase-negative staphylococci, after initial recruitment, six patients were excluded from further analysis; * patients had a single blood culture positive for CNS without any infectious focus.

During the study period, a total of 2,384 patients were screened. Of these, 1,940 patients (81%) presented with less than two criteria for SIRS and 140 patients were excluded due to exclusion criteria prior to participation. After inclusion, six participants were removed from analysis since it was not possible to classify the patient correctly. These patients had a single blood culture positive for CNS without any infectious focus or suffered from invasive mycosis or parasitic infection. A total of 298 study participants showing at least two SIRS criteria were finally analyzed. Figure 1 presents information regarding the recruitment process of study participants.

Bacterial infection was found in 216 patients (72%). Among those patients, the most common infections were blood stream infections (35%) and pneumonia (25%), followed by gastrointestinal system infections (13%) and urinary tract infections (11%). Details on the distribution of ECDC classes of patients with infections are presented in table 1. The most common pathogens isolated from blood cultures were *E. coli* (23%), *S. aureus* (17%), and *K. pneumoniae* (10%). Prior to analysis, five patients with CNS isolated from blood cultures and a focal infection were considered as non-bacteremic infection. SIRS due to causes other than bacterial infection was found in 82 out of the 298 patients (28%). The most frequent causes of SIRS without infection were hematological malignancies (disease or treatment-related, 31%), solid organ malignancies (16%), auto-immune diseases (10%), bleeding or embolism (10%), and cardiomyopathy (9%). The clinical characteristics of the study population are summarized in table 2. Concerning demographic parameters, no significant differences were found between SIRS patients with or without infection or between SIRS patients with or without positive blood cultures. However, SIRS patients with negative blood culture results had a higher rate of antimicrobial treatment prior to the sampling of the blood.

**Table 1:** Summary of infectious foci including ECDC classification of nosocomial infections.

**Type**	**ECDC - class**	**N**	**%**
**Bloodstream infection** ^1^	C-CVC^3^ (n=11), S-DIG^4^ (n=10), S-PUL^5^ (n=8), S-SSI^6^ (n=4), S-SST^7^ (n=3) S-UTI^8^ (n=8), S-OTH^9^ (n=9), S-UO^10^ (n=22)	75	35%
**Pneumonia** ^2^	PN1^11^ (n=4), PN3^12^ (n=5), PN4^13^ (n=4), PN5^14^ (n=40)	53	25%
**Gastrointestinal system infections** ^2^	GI-CDI^15^ (n=3), GI-GE^16^ (n=3), GI-GIT^17^ (n=7), GI-IAB^18^ (n=16)	29	13%
**Urinary tract infection** ^2^	UTI-A^19^ (n=12), UTI-B^20^ (n=12)	24	11%
**Others** ^2^	SYS-CESP^21^(n=4), SYS-DI^22^ (n=3), SSI-S^23^(n=6), SSI-O^24^(n=1), CVS-Card^25^(n=5), CVS-Endo^26^ (n=1), CVS-Vasc^27^(n=1), LRI-Bron^28^(n=2), LRI-Lung^29^(n=2), SST-Skin^30^ (n=3), SST-ST^31^ (n=1), EENT-ORAL^32^ (n=2), CRI1-CVC^33^(n=2), CNS-IC^34^ (n=1), CNS-MEN^35^(n=1)	35	16%
**Total**		216	100%

^1^= blood culture positive ^2^= blood culture negative, ^3^= blood stream infection (BSI), related to central vascular catheter; ^4^= BSI, secondary digestive tract infection; ^5^= BSI, secondary to pulmonary infection; ^6^= BSI, secondary to surgical site infection; ^7^= BSI, secondary to skin and soft tissue infection; ^8^= BSI, secondary to urinary tract infection; ^9^= BSI, secondary to another infection; ^10^= BSI, (confirmed) unknown origin; ^11^= pneumonia, positive quantitative culture from minimally contaminated lower respiratory tract specimen; ^12^= pneumonia, microbiological diagnosis by alternative microbiology method; ^13^= pneumonia, positive sputum culture or non-quantitative culture from lower respiratory tract specimen; ^14^= pneumonia, clinical signs of pneumonia without positive microbiology; ^15^= gastrointestinal system infections (GI) *clostridium difficile* infection; ^16^= GI, gastroenteritis (excluding CDI); ^17^= GI, gastrointestinal tract (oesophagus, stomach, small and large bowel, and rectum), excluding GE, CDI; ^18^= GI, Intra-abdominal, not specified elsewhere; ^19^= urinary tract infection (UTI), microbiologically confirmed symptomatic UTI; ^20^= UTI, not microbiologically confirmed symptomatic UTI; ^21^= systemic infections (SYS), clinical sepsis in adults and children; ^22^= SYS, disseminated infection; ^23^= surgical site infection (SSI), superficial; ^24^=SSI, organ/space; ^25^= cardiovascular system infection (CVS), myocarditis or pericarditis; ^26^= CVS, endocarditis; ^27^= CVS, arterial or venous infection; ^28^= lower respiratory tract infection, other than pneumonia (LRI), bronchitis, tracheobronchitis, bronchiolitis, tracheitis, without evidence of pneumonia; ^29^= LRI, other infections of the lower respiratory tract; ^30^= skin and soft tissue infections (SST), skin; ^31^= SST, soft tissue (necrotising fascitis, infectious gangrene, necrotizing cellulitis, infectious myositis, lymphadenitis, or lymphangitis); ^32^= eye, ear, nose or mouth infection (EENT), oral cavity (mouth, tongue, or gums); ^33^= central vascular catheter-related infection (CRI), general CVC-related infection (no positive blood culture); ^34^= central nervous system infection (CNS), intracranial infection; ^35^= CNS, meningitis or ventriculitis

**Table 2:** Patients characteristics and demographic data of the study population.

**Parameter**	**Overall**	**Inflammation**	**Infection**	**p-values**	**Non-bacteremic**	**Bacteremic**	**p-values**
**Number**	298	82 (28%)	216 (72%)		223 (75%)	75 (25%)	
**Age**	58.0 (43.0-70.0)	58.0 (39.8-67.0)	60.0 (45.0-71.0)	0.127	58.0 (43.0-69.0)	61.0 (45.0-71.0)	0.291
**Male**	173 (58%)	48 (59%)	125 (58%)	>0.999	143 (64%)	39 (52%)	0.227
**Body mass index** ^1^	24.8 (21.6-28.1)	25.4 (21.9-30.1)	24.5 (21.5-27.7)	0.178	25.0 (21.6-28.4)	24.4 (21.5-27.1)	0.220
**Length of hospital stay**	16.0 (9.0-28.0)	19.0 (9.0-29.3)	15.0 (9.0-27.8)	0.386	15.0 (9.0-27.0)	19.0 (10.0-28.0)	0.520
**In-hospital morality**	33 (11%)	10 (12%)	33 (15%)	0.684	26 (12%)	7 (9%)	0.674
**2 SIRS-symptoms**	120 (40.3%)	29 (24.2%)	91 (75.8%)	0.443	93 (77.5%)	27 (22.5%)	0.429
**3 SIRS-symptoms**	128 (43.0%)	40 (31.2%)	88 (68.8%)	0.444	96 (75%)	32 (25%)	0,440
**4 SIRS-symptoms**	50 (16.8%)	13 (26%)	37 (74%)	0.556	34 (68%)	16 (32%)	0.219
**Squeeze**	149 (50%)	36 (43%)	113 (52%)	0.243	100 (45%)	49 (65%)	0.003
**Alteration in mental status**	29 (10%)	6 (7%)	23 (11%)	0.672	18 (8%)	11 (15%)	0.203
Antibiotics before onset*	15/283	5/77	10/206	0.163	14/209	1/74	0.008

^1^body mass index, SIRS = Systemic Inflammatory Response Syndrome; numeric values are given as median (Q_1_-Q_3_) *yes/no or undocumented.

### Prediction of infection

**Figure 2:**
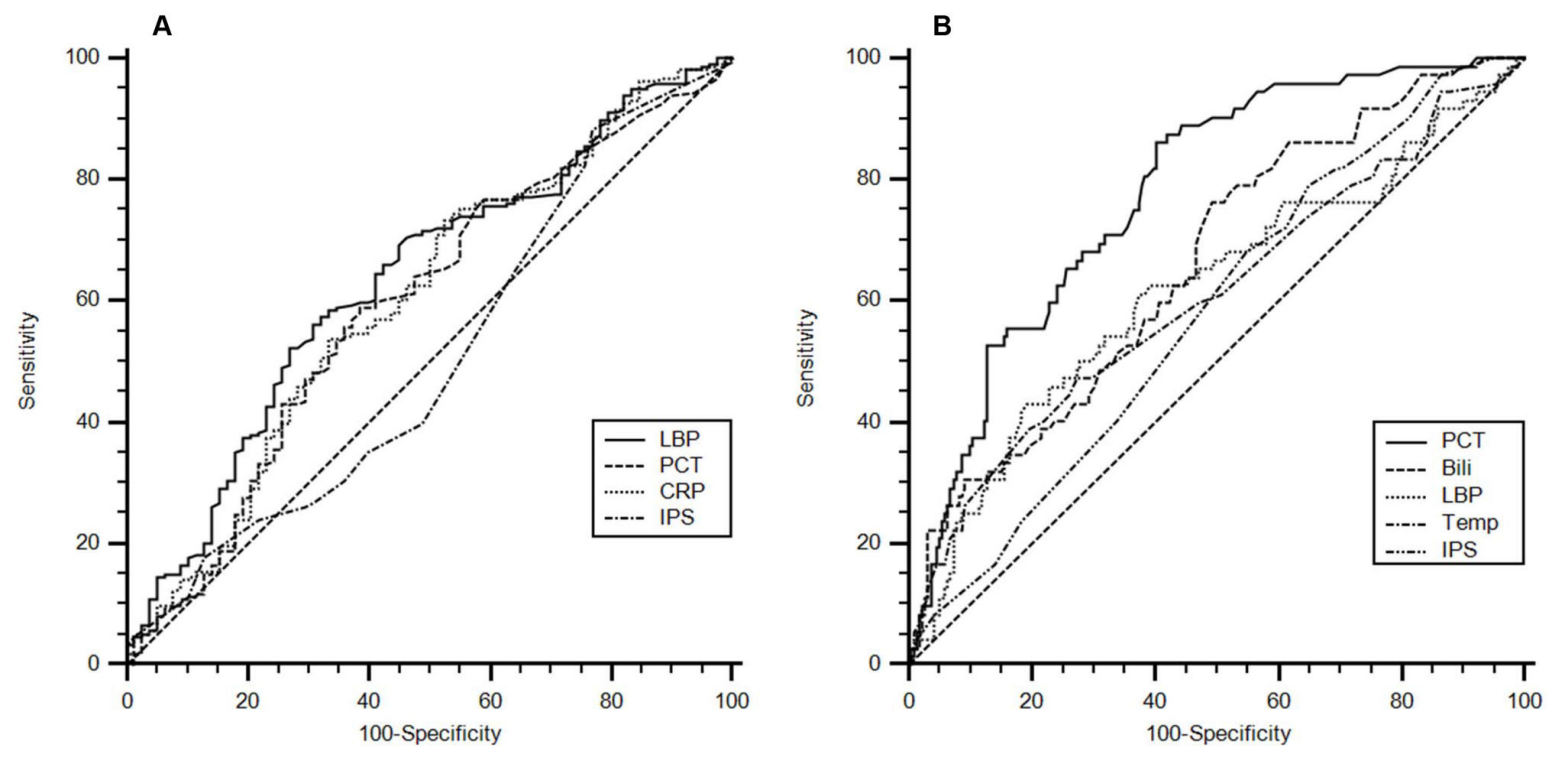
ROC-curves of various parameters. A: prediction of infection; ROC-AUCs of LBP: 0.63, PCT: 0.59, CRP: 0.59, IPS: 0.51;. B: prediction of bacteremia; ROC-AUCs of PCT: 0.78, Bili (bilirubin): 0.65, LBP: 0.62, Temp (body temperature): 0.61, IPS: 0.58;.

The median IPS among SIRS patients with infection was 16 and was thus not different to the median IPS observed in SIRS patients without infection (p = 0.769). The area under the ROC curve was 0.51 (see: Figure 2A), yielding 30.1% sensitivity, 64.6% specificity, 26.0% NPV, and 69.2% PPV. After application of the Bonferroni-Holm method, none of the individual parameters forming the IPS demonstrated a significant difference between both groups. The most discriminatory marker was CRP (p = 0.017) with an ROC-AUC of 0.59. Information on the capacity of the individual IPS parameters and sepsis biomarker to discriminate between infection and inflammation in SIRS patients is provided in tables 3 and 4.

**Table 3:** Discriminatory capacities of parameters for infection.

**Parameter**	**Overall**	**Inflammation**	**Infection**	***p*-value**	**ROC-AUC**
**LBP**	24.9 (15.6-37.2)	19.1 (13.6-29.3)	26.2 (17.5-26.5)	0.001*	0.63 (0.57-0.68)
**PCT**	0.4 (0.1-1.8)	0.3 (0.1-1.2)	0.5 (0.2-2.2)	0.014	0.59 (0.53-0.65)
**CRP**	14.5 (9.3-21.6)	12.8 (7.2-20.1)	15.4 (10.5-21.8)	0.017	0.59 (0.53-0.65)
**SOFA**	1.0 (0.0-3.0)	2.0 (0.8-3.0)	1.0 (0.0-3.0)	0.053	0.57 (0.51-0.63)
**Temp** ^1^	38.5 (38.0-38.9)	38.3 (37.5-38.8)	38.5(38.1-39.0)	0.093	0.56 (0.50-0.62)
**IL-6**	48.5 (28.4-105.3)	39.9 (22.0-105.2)	52.3 (30.6-107.3)	0.116	0.56 (0.50-0.62)
**RR** ^2^	21.0 (16.0-24.0)	21.0(16.0-24.0)	21.0 (16.0-24.0)	0.274	0.54 (0.48-0.60)
**HBR** ^3^	98.0 (91.0-107.0)	97.5 (91.0-104.0)	100 (90.0-109.0)	0.392	0.53 (0.47-0.59)
**WBC**	9.6 (5.4-13.5)	8.8 (2.8-14.5)	9.7 (5.8-13.3)	0.435	0.53 (0.47-0.59)
**IPS**	16.0 (11.0-17.0)	16.0 (11.0-18.3)	16.0 (11.0-17.0)	0.769	0.51 (0.45-0.57)
**Bilirubin**	0.7 (0.5-1.1)	0.7 (0.5-1.0)	0.7 (0.5-1.1)	0.848	0.51 (0.45-0.57)

numbers represent the median (Q_1_-Q_3_); 95% confidence interval of the ROC-AUC is given in parentheses; parameters are ranked in the order of their p-values; ^1^body temperature, ^2^respiration rate, ^3^heart beat rate.

**Table 4:** Performance measures of the IPS and sepsis biomarkers with statistical significance.

**Outcome**	**Parameter**	**Sensitivity**	**Specificity**	**NPV**	**PPV**
**Sepsis**	IPS	30.1 (24.0-36.7)	64.6 (53.3-74.9)	26.0 (20.1-32.6)	69.2 (58.8-78.3)
	LBP	57.5 (50.6-64.2)	67.1 (55.6-77.3)	36.8 (28.9-44.2)	82.6 (75.5-88.3)
**Bacteremia**	IPS	21.3 (12.7-32.3)	65.0 (58.4-71.3)	71.1 (64.3-77.2)	17.0 (10.1-26.2)
	Temp^1^	48.0 (36.3-59.9)	72.2 (65.8-78.0)	80.5 (74.3-85.8)	36.7 (27.2-47.1)
	PCT	86.3 (76.3-93.2)	59.6 (52.7-66.1)	92.9 (87.3-96.6)	41.5 (33.5-49.7)
	Bilirubin	79.7 (68.8-88.2)	46.2 (39.5-53.0)	87.3 (79.9-92.7)	33.0 (26.1-40.4)
	LBP	61.6 (49.5-72.8)	62.3 (55.5-68.7)	83.0 (76.4-88.4)	35.2 (26.9-44.1)

^1^ body temperature, 95% confidence interval is given in parentheses.

Among the biomarkers evaluated in the present study, the best parameter was LBP with a median level of 26.2 pg/ml in patients with infections and a median level of 19.1 pg/ml in those without infections. Thus, LBP was significantly higher in SIRS patients with infection when compared with the LPS of SIRS patients without infection (p = 0.001). Although the ROC-AUC was in a moderate range (0.63), it was significantly higher compared to the ROC-AUC of the IPS (DeLong test, p = 0.043). Using a cut-off value of 24.35 pg/ml (Youden-index method), LBP demonstrated 57.5% sensitivity, 67.1% specificity, 36.8% NPV, and 82.6% PPV. Concerning the other sepsis biomarkers evaluated, no significant differences were observed after correcting for errors related to multiple testing.

### Prediction of bacteremia

Regarding the utility of the IPS to predict bacteremia (n=75, 25%) in patients with SIRS, no differences were observed between the IPS values among patients with bacteremia when compared to those patients with negative blood culture results. Details are presented in tables 4 and 5. The ROC-AUC of the IPS was 0.58 (see figure 2B), the sensitivity and specificity were 21.3% and 65.0% respectively, with 71.1% NPV and 17.0% PPV. Among the individual parameters of the IPS, significant differences were found for bilirubin (forming the SOFA score for the IPS) as well as for body temperature. Bacteremic patients had significantly increased serum levels of bilirubin (0.82 mg/dl) when compared with SIRS patients without bacteremia (0.64 mg/dl, p <0.0001), with a ROC-AUC of 0.65. Using a cut-off value of 0.61 mg/dl, bilirubin resulted in 79.7% sensitivity, 46.2% specificity, 87.3% NPV, and 33.0% PPV. Moreover, patients with bacteremia had increased body temperature compared to non-bacteremic SIRS patients (38.5°C vs. 38.4°C, p = 0.004), with a ROC-AUC of 0.61. Using 38.6°C as a cut-off value, the assessed sensitivity was 48.0% with 72.2% specificity, 80.5% NPV, and 36.7% PPV.

**Table 5:** Discriminatory capacities of parameters for bacteremia.

Parameter	Overall	Non- bacteremic	Bacteremic	*p*-value	ROC-AUC
PCT	0.4 (0.1-1.8)	0.3 (0.1-1.1)	2.5 (0.4-8.7)	<0.001*	0.78 (0.72-0.83)
Bilirubin	0.7 (0.5-1.1)	0.6 (0.5-0.9)	0.8 (0.6-1.7)	<0.001*	0.65 (0.59-0.71)
LBP	24.9 (15.6-37.2)	23.0 (15.6-35.3)	30.4 (19.6-44.5)	0.003*	0.62 (0.56-0.67)
Temp^1^	38.5 (38.0-38.9)	38.4 (37.9-38.8)	38.5 (38.1-39.2)	0.004*	0.61 (0.55-0.67)
HBR^2^	98.0 (91.0-107.0)	97.0 (90.0-105.0)	100.0 (92.0-110.0)	0.020	0.59 (0.53-0.65)
IPS	16.0 (11.0-17.0)	16.0 (10.0-17.0)	16.0 (14.0-18.0)	0.033	0.58 (0.52-0.64)
SOFA	1.0 (0.0-3.0)	1.0 (0.0-3.0)	2.0 (0.0-4.0)	0.050	0.57 (0.52-0.63)
CRP	14.5 (9.3-21.6)	14.2 (8.7-21.9)	15.8 (11.4-21.4)	0.139	0.56 (0.50-0.61)
RR^3^	21.0 (16.0-25.0)	21.0 (16.0-24.0)	21.0 (18.0-25.0)	0.144	0.56 (0.50-0.61)
IL-6	48.5 (28.4-105.3)	48.0 (26.5-99.2)	53.7 (33.4-115.3)	0.297	0.54 (0.48-0.60)
WBC	9.6 (5.4-13.5)	9.7 (5.4-13.5)	9.5 (5.5-13.8)	0.936	0.50 (0.44-0.56)

numbers represent the median (Q_1_-Q_3_); 95% confidence interval of the ROC-AUC is given in parentheses; parameters are ranked in order of their p-values; ^1^body temperature, ^2^heart beat rate, ^3^respiration rate.

Among the biomarkers evaluated, PCT was the best discriminator between SIRS patients with and without bacteremia. The median PCT value among patients with bacteremia was 2.5 ng/ml and thus significantly higher in comparison with a median of 0.3 ng/ml found in patients without bacteremia (p <0.0001). The ROC-AUC was 0.78, which was found to be superior compared to other assessed biomarkers. For ROC-AUC comparison between PCT and other assessed parameters, the DeLong test was applied, resulting in a p-value range between <0.001 and 0.0085. A cut-off value of 0.35 ng/ml was computed, resulting in 86.3% sensitivity, 59.6% specificity, 92.9% NPV, and 41.5% PPV. Statistical significance was also found for LBP with higher values in patients with bacteremia compared to patients without bacteremia (23.0 vs. 30.4 pg/ml, p = 0.003). The ROC-AUC for LBP was 0.62 with 61.6% sensitivity, 62.3% specificity, 83.0% NPV, and 35.2% PPV. No significant differences were assessed in IL-6, CRP, or WBC (table 5).

## Discussion

In patients with SIRS, detection of infection is crucial for proper management. Since there is a lack of accurate, rapid and cost efficient diagnostic tools for the identification of septic patients, physicians are regularly faced with resulting uncertainties [22]. Moreover, there is major variation in the host´s immune response. The spectrum of the host´s immune response ranges from immunoparalysis to hyperinflammation, partly independent of the expansion of the infectious focus. Therefore, the robustness of biomarkers is pivotal for their applicability in the everyday routine [23,24].

The IPS and various sepsis biomarkers have been shown to be beneficial in the identification of infection, although the data on its clinical utility is controversial. Most studies have been conducted in critical care patients with severe disease or at emergency departments, but evaluation in standard care patients with an appropriate pre-selection in order to focus on relevant patients has rarely been performed. In addition, in the majority of studies outcome parameters were based on discharge diagnosis rather than on well evaluated and reproducible criteria.

Due to the absence of a real gold standard and a lack of an applicable SIRS classification system, surveys on patients with suspected infection are challenging. In the present study, 2,384 standard care patients with clinical suspicion of infection were consecutively screened for the occurrence of SIRS. To obtain a relevant study population, only patients with SIRS were included. IPS and sepsis biomarkers were evaluated regarding their potency to differ between SIRS patients with infection and those with SIRS due to other causes. Furthermore, the capacity to identify SIRS patients with bacteremia was assessed. Infection as the main outcome parameter was defined according to an established and robust protocol [21]. In order to minimize false positive blood culture results, patients with a possible contaminant in their blood culture and an unclear infectious focus were excluded [19,20].

Regarding the differentiation of SIRS patients with infection from those with systemic inflammation due to other reasons, the diagnostic ability of the IPS and sepsis biomarkers was poor in the present study. In fact, the IPS was developed as an infection score in severely ill patients, for which Bota et al. have shown a high NPV (89.5%) to exclude infections [11]. Likewise, other initial evaluations in severely ill patients as well as in hemato-oncological patients were promising [25,26]. In contrast to these studies, only patients at risk of infection were included in the current study, as described above. This pre-selection step led to an increased prevalence of infection and subsequently to an increased pre-test probability. The poor outcome of the IPS might be related to this alteration of the prevalence of infection, indicating low robustness of the score.

For prediction of infection in SIRS patients, no parameter displayed persuasive discriminatory capacities. Of the sepsis biomarkers, in the present study LBP was the most reliable parameter with its ROC-AUC as well as sensitivity and specificity remaining in a moderate range. According to our data, its clinical relevance regarding this differentiation setting must be questioned. However, in literature, LBP presents a better predicting power to identify infection or sepsis compared to our study [27,28]. Those studies also included patients without SIRS and were conducted at intensive care units. PCT and CRP initially showed discriminatory capacities, but were not considered to differentiate significantly after applying the Bonferroni-Holm correction for multiple testing. Their ROC-AUC curves were also in a lower range. Likewise, in other studies PCT and CRP present a better diagnostic potency compared to our study [13,28].

Regarding the prediction of bacteremia, the IPS and most of the sepsis biomarkers applied demonstrated better diagnostic abilities compared to the prediction of infection. However, after applying the Bonferroni-Holm correction, the IPS was not found to reveal statistically different results. Among its individual clinical parameters, body temperature was the best predictor of bacteremia. Nevertheless, the relevance of the temperature difference (0.1° Celsius) in SIRS patients with and without bacteremia must be questioned. 

Of interest, serum bilirubin, a parameter which was analyzed to compute the IPS, presented a significant difference between patients with and without bacteremia. This finding is described in literature [29,30,31]. Hyperbilirubinemia is a risk factor, as well as a recognized complication of sepsis, which is associated with a reduction of the bile flow in hepatocytes [32,33]. To our knowledge, a systemic analysis of bile acid flow in patients with severe infections has not yet been assessed, although in 1901 Osler already described *toxaemic jaundice* in patients with pneumonia [34]. 

Among the sepsis biomarkers evaluated in the present study, PCT was the best parameter for the prediction of bacteremia. Secondarily, LBP, which was also associated with bacteremia [35,36], presented a lower diagnostic performance compared to PCT, with a ROC-AUC in a moderate range. The superiority of PCT related to other parameters is in accordance with the literature [37,38]. In recent studies, conducted at an emergency department, similar ROC curve results for the prediction of bacteremia were assessed [39,40]. In a meta-analysis including 30 studies altogether involving 3,244 patients with suspected sepsis, a mean sensitivity of 77%, and specificity of 79% for the prediction of bacteremia was shown for PCT [41]. It is noteworthy that, of the 30 studies included, only two studies had been conducted in standard care wards [42,43]. In both studies, the ROC-AUC of PCT was in a comparable range (both: 0.75).

Interestingly, widely used parameters such as CRP or WBC showed low discriminatory capacity to differentiate between systemic inflammation based on infection or other causes or bacteremia and non-bacteremia in patients with SIRS. The usefulness of CRP is the subject of some controversy [13,39,44,45,46]. Accordingly, our data suggest the routine use of CRP as a standard infection marker should be reconsidered.

Due to the complexity of the inflammatory response elicited by infectious or non-infectious stimuli, it is most likely that a combination of biomarkers is needed to improve diagnostic abilities [47]. In our study, multivariate modeling performed with logistic regression did not improve the predictive value of several parameters (data not shown). This is in accordance with Tramp et al., who reported that a combination of various biomarkers or clinical signs did not improve the diagnostic ability of PCT regarding bacteremic patients. In this survey, PCT had a sensitivity of 89%, a specificity of 58%, and a ROC-AUC of 0.80 [39]. It is likely that non-linear prediction models, including support vector machines or artificial neural networks, are be better suited for this classification task and might improve the diagnostic ability of combined analysis of parameters [48,49,50].

Low robustness might explain the lower discriminatory power of biomarkers and especially of the IPS in contrast to previous results. In IPS studies, no pre-selection of cases was done, leading to a lower pre-test probability and subsequently to a higher NPV. Therefore, our findings emphasize the need for careful validation in different patient populations [51]. In this survey a cohort study design was chosen, including only patients fulfilling two or more SIRS criteria. This leads to a higher prevalence of infection and bacteremia and subsequently to a higher pre-test probability than described elsewhere.

### Limitations

First, for screening of potential study participants the primary selection criteria was blood culture request by the physician in charge was used as primary selection criterion. Therefore, a possible selection bias cannot be excluded. Secondly, biomarker samples were obtained within a time frame of 18 hours after the blood culture request, which might imply a time-dependent variation in cytokine patterns. In our opinion this does not represent a major limitation, since biomarkers, including LBP and CRP, were found to be the highest on the third day after the onset of sepsis [52]. Further, the diagnostic ability of PCT was in the range of similar studies. Moreover, antibiotic pre-medication prior to the taking of blood cultures was more frequently found in patients without bacteremia, which might have led to false negative cases. However, antibiotic pre-medication is a dilemma in everyday routine and therefore was not found to be a major limitation. Additionally, the blood culture flasks used contained resin particles, which were shown to eliminate or decrease the concentration of antimicrobial substances present in inoculated blood [53].

## Conclusions

In this patient cohort consisting of standard care ward patients with SIRS, the prevalence of infection was 72%. To correctly classify the remaining 28% SIRS patients, a conclusive definition of SIRS is needed to improve the quality of such studies. The IPS and widely used biomarkers, including CRP and WBC, showed a low diagnostic performance regarding the identification of infection and bacteremia. The diagnostic abilities of sepsis biomarkers performed in a similar way. Among the sepsis parameters tested, no parameter had persuasive diagnostic capacities to identify infections in patients with SIRS. For the prediction of bacteremia, PCT was the most promising parameter. Furthermore, a change in the bilirubin pattern could indicate ongoing infection. Thus, bilirubin might be useful as a cheap screening parameter for identification of patients at risk of suffering from bacteremia. A linear combination of several parameters did not improve the diagnostic ability of PCT. Non-linear models are probably more appropriate for this classification task. 
